# Laminin-411 Is a Vascular Ligand for MCAM and Facilitates TH17 Cell Entry into the CNS

**DOI:** 10.1371/journal.pone.0040443

**Published:** 2012-07-06

**Authors:** Ken Flanagan, Kent Fitzgerald, Jeanne Baker, Karin Regnstrom, Shyra Gardai, Frederique Bard, Simonetta Mocci, Pui Seto, Monica You, Catherine Larochelle, Alexandre Prat, Samuel Chow, Lauri Li, Chris Vandevert, Wagner Zago, Carlos Lorenzana, Christopher Nishioka, Jennifer Hoffman, Raquel Botelho, Christopher Willits, Kevin Tanaka, Jennifer Johnston, Ted Yednock

**Affiliations:** 1 Elan Pharmaceuticals, San Francisco, California, United States of America; 2 Neuroimmunology Research Laboratory, Center of Excellence in Neuromics, CRCHUM, Université de Montréal, Montréal, Quebec, Canada.; University of Muenster, Germany

## Abstract

TH17 cells enter tissues to facilitate pathogenic autoimmune responses, including multiple sclerosis (MS). However, the adhesion molecules involved in the unique migratory capacity of TH17 cells, into both inflamed and uninflamed tissues remain unclear. Herein, we characterize MCAM (CD146) as an adhesion molecule that defines human TH17 cells in the circulation; following in vitro restimulation of human memory T cells, nearly all of the capacity to secrete IL-17 is contained within the population of cells expressing MCAM. Furthermore, we identify the MCAM ligand as laminin 411, an isoform of laminin expressed within the vascular endothelial basement membranes under inflammatory as well as homeotstatic conditions. Purified MCAM-Fc binds to laminin 411 with an affinity of 27 nM, and recognizes vascular basement membranes in mouse and human tissue. MCAM-Fc binding was undetectable in tissue from mice with targeted deletion of laminin 411, indicating that laminin 411 is a major tissue ligand for MCAM. An anti-MCAM monoclonal antibody, selected for inhibition of laminin binding, as well as soluble MCAM-Fc, inhibited T cell adhesion to laminin 411 *in vitro*. When administered in vivo, the antibody reduced TH17 cell infiltration into the CNS and ameliorated disease in an animal model of MS. Our data suggest that MCAM and laminin 411 interact to facilitate TH17 cell entry into tissues and promote inflammation.

## Introduction

A subset of CD4+ T cells, termed TH17 cells, has been implicated in the pathogenesis of a number of autoimmune diseases, including multiple sclerosis (MS) and its associated animal model, experimental autoimmune encephalomyelitis (EAE; [1], [2]). Much attention on the enhanced pathogenicity of TH17 cells has focused on their ability to secrete a distinct subset of cytokines, including IL-17 and IL-22. However, the specific role of TH17 cytokines has been called into question, since a conditional knockout of IL-17 is insufficient to eliminate EAE progression, [3], [4] suggesting that the role of TH17 cells is more complex than production of a particular cytokine by itself.

IL-17 producing cells are enriched within the CCR6+ population of CD4+ T cells, likely conferring a unique migration pattern throughout the vasculature [5]. CCR6 expression on T cells is required for migration into the CNS and the progression of EAE. It has been proposed that a potential site for CCR6-dependent T cell entry into the CNS is the choroid plexus due to its constitutive expression of CCL20, a known ligand of CCR6. Multiple adhesive pathways are involved in chronic inflammation, including α4 integrin, which is expressed broadly on all T cells subsets, as well as B cells and monocytes. In the case of α4 integrin, findings made in EAE have been clinically validated with a therapeutic antibody in MS [6], [7], [8]. However, specific adhesive pathways have not been described to help explain the unique migratory properties of TH17 cells and their potent ability to initiate or propagate an inflammatory response.

Herein we report the identification of Melanoma cell adhesion molecule (MCAM) as an adhesion receptor on TH17 cells in the circulation, and we identify a major tissue ligand for MCAM as laminin 411, which is expressed selectively by vascular endothelial cells. Our results with MCAM expression on TH17 cells independently confirms and extends a recent report by Dagur, et al., and is consistent with previous reports that MCAM supports cell interactions within the vasculature [9], [10], [11], [12]. Our novel results with laminin 411 offers insight into how MCAM on TH17 cells may function within the adhesion cascade.

We identified MCAM on TH17 cells in a microarray study aimed at discovering novel molecules associated with IL-17 production in CD4+ T cells. Linked with tumor cell adhesion and metastasis, MCAM seemed a possible candidate to augment the tissue-infiltrative potential among T cells [13]. MCAM is enriched on T cell clones generated from MS patients, and MCAM expressing T cells are particularly prominent at sites of inflammation [11], [14]. We found that isolation of MCAM expressing CD4+ memory T cells from the circulation strongly enriched for expression of IL-17. Furthermore, when sorted and stimulated *in vitro*, nearly all IL-17 production from circulating memory CD4+ T cells emanated from MCAM expressing T cells, supporting the idea that MCAM expression is a defining characteristic of TH17 cells. Similarly, production of the TH17 cytokines IL-22 and CCL20 was strongly enriched within the population of MCAM expressing T cells, while IFNγ (TH1 cytokine) was produced by a similar percentage of both MCAM+ and MCAM− cells. Though previous data suggested that murine T cells do not express MCAM, we found that such a population could be generated, specifically under TH17 polarizing conditions.

It has been reported that MCAM functions as a homophilic adhesion molecule, however to the best of our knowledge, however direct biochemical evidence for homophilic interaction with the purified receptor is lacking, and is complicated by the fact that MCAM expression in cells induces microvilli, which enhance cell adhesion through independent pathways [12], [15]. Using multiple approaches with defined molecular systems we could not measure an interaction of MCAM with itself, including MCAM-dependent cell adhesion to MCAM-expressing cells, recombinant MCAM interaction with MCAM on the cell surface, nor of the recombinant receptor with itself. These results suggest that homophilic interactions may be limited to specific cell types, to particular experimental conditions, or dependent upon other undefined factors, as has been suggested previously [16]. In an unbiased approach, we set out to identify other potential ligands for MCAM by examining the interaction of purified soluble MCAM-Fc with tissues. Mouse and human MCAM-Fc bound with high selectivity and strong definition to the endothelial basement membrane of mouse blood vessels, and we were able to identify the specific ligand as laminin 411, an isoform of laminin that is expressed along the vascular endothelium. Laminin 411 has already been implicated in the migration of murine T cells into the CNS, making this finding of particular interest since the immune cell adhesion receptor was not defined [17], [18]. We validated the laminin 411/MCAM interaction using purified recombinant molecules and surface plasmon resonance, and confirmed its relevance and specificity by looking at tissue from mice with targeted deletion of laminin 411. Further, we developed a monoclonal antibody that blocks the interaction of mouse MCAM with laminin 411 and demonstrated that it blocks T cell adhesion to laminin 411 *in vitro*, reduces TH17 cell entry into the CNS in vivo, and decreases disease associated with EAE.

We thus propose that MCAM and laminin 411 contribute to the unique migratory capability of TH17 cells. The broad expression of laminin 411 in the vasculature of both mice and humans supports the idea that MCAM expressing T cells could function in immune surveillance of healthy tissue and/or propagation of an inflammatory response.

## Methods

### Isolation, characterization and polarization of human T cells

Buffy coats were obtained from healthy human donors (Stanford Blood Center, Palo Alto, CA) and CD4 T cells were negatively enriched using RosetteSep (Stem Cell Technologies) and manufacturer's protocols. Where indicated, CD4+CD45RO+ memory T cells were further negatively purified using magnetic beads (Miltenyi Biotec). T cells were plated (2×10^5^ cells/well) in anti-CD3 (5 µg/ml, BD Pharmingen) coated 96 well U bottom plates in RPMI containing 10% heat-inactivated FCS (HyClone Laboratories), penicillin, streptomycin, L-glutamine, anti-IFNγ (5 ug/ml; R&D Systems), anti-IL4 (0.5 ug/ml, R&D Systems) and anti-CD28 (2 µg/ml; BD Pharmingen) for five days. Where indicated, TGFβ (2 ng/ml), IL12, IL1β, and/or IL-23 (all at 20 ng/ml) were added. All cytokines were obtained from R&D Systems.

### RNA isolation and microarray

For microarray experiments, human CD4+ T cells were isolated as above, stained for CD161 and CCR6 (both from BD Pharmingen) and sorted into CD4+CD161−CCR6− (non-TH17) and CD4+CD161+CCR6+ (TH17) cells from three independent healthy donors. RNA was isolated from half of the cells from each donor immediately (circulating) and the other half was stimulated with plate bound anti-CD3 and soluble anti-CD28 as above, in the absence of exogenous cytokines for four days (activated) before RNA isolation. RNA was amplified (Nugen) and hybridized on Human U133 Plus 2.0 Array (Affymetrix) at 45°C. All microarray performed at Expression Analysis (Durham, NC). For determination of IL-17 concentrations in the supernatant, ELISA was performed using a commercial kit (R&D Systems).

### Immunofluorescent staining

Mouse tissues were snap frozen in OCT and sectioned at 10 *u*M. Tissues from LAMA4−/− mice [19] were a generous gift from Lydia Sorokin (University of Muenster, Germany). Sections were fixed in cold acetone for 10 minutes, blocked and stained with directly conjugated anti-pan-laminin (Novus Biologicals), MCAM-Fc, anti-CD31 (BD Pharmingen) or anti-laminin α4 (R&D Systems). All staining reagents were directly conjugated, and incubated overnight at 4°C at 0.5 µg/mL unless otherwise indicated.

### Laminin binding assay

Parental CHO cells, lacking MCAM expression, or CHO cells stably transfected with mouse MCAM (CHO.mMCAM) were incubated for 30 minutes in the presence of recombinant laminin 411 or recombinant laminin 511 (20 µg/mL, Biolamina, Stockholm, Sweden) at 37°C [20]. Cells were washed thoroughly and laminin binding to the surface of cells was detected with fluorescently labeled pan laminin antibody (Novus Biologicals) by flow cytometry. Where indicated, cells were preincubated for 15 minutes in the presence of 10 µg/mL neutralizing anti-mouse MCAM.

### Cell adhesion assay

Recombinant laminins were coated at 20 µg/mL in PBS into 96 well plates overnight at 4°C as per manufacturer's protocols, and washed three times before blocking with 2% BSA for one hour. Human CD4+CD45RO+ memory T cells were cultured for five days in the presence of TGFβ and IL1β (∼50% MCAM+) and 4×10^4^ were added to plates. Following incubation, cells were washed and remaining cells stained with crystal violet, solubilized in 1% SDS and absorbance was read at 550 nm.

### LAMA4−/− mice

LAMA4−/− mice were generated in the laboratory of Karl Tryggvason [19], and tissues were kindly provided by Lydia Sorokin [18].

### Modified Stamper Woodruff assay [21]

MOLT4 cells (ATCC) were labeled for 30 minutes with 1 µM calcein for 30 minutes, washed and resuspended at 5×10^6^ cells/mL. Aliquots of 100 µL were added to 10 µM acetone fixed serial sections of healthy mouse brain and incubated at 37°C for 45 minutes with constant rotation. Sections were gently washed, mounted in DAPI containing mounting medium and visualized by fluorescent microscopy.

### Mouse T cell polarization and EAE experiments

All EAE experiments were performed at Hooke Laboratories (Lawrence, MA). Nine week old female SJL mice (Charles River Laboratories) were immunized using EAE induction kit (Hooke Laboratories) following manufacturer's protocol after acclimation in the laboratory for 3 weeks. In summary, mice received a subcutaneous injection of CFA emulsion containing 200 ug with PLP_139–151_ per mouse at four sites in the back. Two sites of injection were in the area of the upper back, approximately 1 cm caudal of the neck line. The other two sites were in the area of the lower back, approximately 2 cm cranial of the base of the tail. Injection volume was 0.05 mL at each site.

In experiments to analyze the effects of polarization conditions on MCAM expression, splenocytes from immunized mice were isolated on day 11, and cultured in the presence of PLP (5 µg/mL, Hooke Laboratories). Cells were cultured for five days in RPMI containing 10% heat-inactivated FCS (HyClone Laboratories), penicillin, streptomycin, L-glutamine, anti-IFNγ (5 ug/ml; R&D Systems), anti-IL4 (0.5 ug/ml, R&D Systems) and β-ME (50 µM). Where indicated, human TGFβ (5 ng/ml) and/or murine IL-23 (20 ng/mL), and/or murine IL-1β (20 ng/mL) was added. All cytokines were from R&D Systems. Cells were stained with anti-CD4, (BD Pharmingen) and anti-mMCAM.

For EAE studies, immunized mice were randomized into groups based on clinical scores on day of onset. On the second day following disease onset, mice were treated (N = 15 per group) i.p. with either neutralizing rat anti-MCAM (clone 15) or isotype control (Bioxcell) at 10 mg/kg body weight, and every day thereafter. Mice were monitored daily and scored for in a blinded manner, and body weights were obtained every 2–3 days.

## Results

### IL-17 production is enriched within the circulating population of human memory T cells expressing MCAM

To identify novel targetable molecules associated with the unique tissue-infiltrative capacity of TH17 cells, we sorted peripheral blood lymphocytes based on surface receptors known to be enriched on TH17 cells. Importantly, this approach had the benefit of purifying T cells directly from the circulation that have the potential to produce IL-17, while maintaining their resting state.

Surface expression of both CCR6 and CD161 have been shown to predict the ability of a CD4 T cell to produce IL-17 [22], [23], and in support of our approach to isolate TH17 cells, we confirmed that most of the IL-17 production within CD4 T cells was contained within the CCR6+CD161+ sorted population (data not shown). Circulating CD4+ T cells from three healthy donors were sorted into CCR6+CD161+ and CCR6−CD161− cells. Half of the cells from each donor were immediately used for RNA isolation, while RNA was isolated from the other half following activation for four days in the presence of anti-CD3 and anti-CD28 (without exogenous cytokines). We identified the TH17 transcription factor *Rorc* solely within the enriched TH17 population, and IL-17 message induction was exclusive to the group of activated TH17 cells (data not shown). Since these TH17 genes, and others, sorted to appropriate groups, our strategy to identify genes associated with TH17 was sufficient to identify additional, novel genes with similar expression patterns. One such gene, MCAM, was identified with expression nearly exclusive to the circulating TH17 cells.

**Figure 1:**
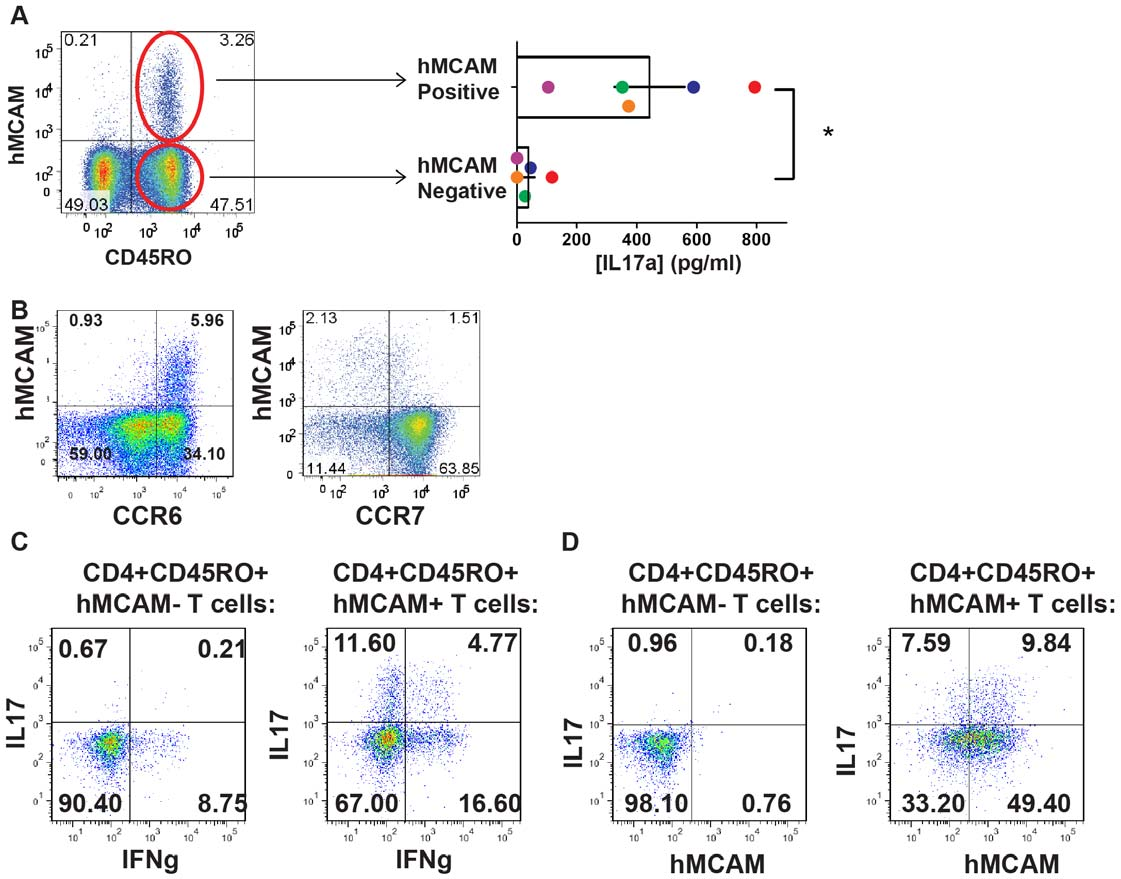
hMCAM identifies a small population of human memory T cells with the majority of the capacity to secrete IL-17. (A) hMCAM is expressed on about 3–6% of circulating human CD4+ T cells, nearly exclusively in the CD45RO+ memory T cell pool. CD4+CD45RO+ memory T cells from five individual donors were further sorted into hMCAM− and hMCAM+ populations and stimulated for four days in the presence of anti-CD3 and anti-CD28 and supernatants were analyzed for IL-17. Each individual donor (n = 5) is represented by a different color (* indicates p<0.05). (B) Freshly isolated human CD4+ T cells were stained for hMCAM and coexpression of chemokine receptors, CCR6 and CCR7. (C) Human CD4+CD45RO+ T cells were sorted based on hMCAM expression, and stimulated with anti-CD3 and anti-CD28 in the absence of exogenous cytokines. Cells were collected after five days, following PMA/Ionomcycin stimulation with golgi inhibition for the final five hours. Cells were stained for intracellular IL17 and IFNγ. (D) Cells were sorted for hMCAM expression and stimulated as in C. They were then restained for MCAM expression, as well as intracellular IL17.

From separate blood samples, we confirmed surface hMCAM expression on a small population of CD4+ T cells (typically 3–6% from healthy donors), that existed almost entirely within the CD45RO+ memory population of T cells (**Figure 1A, left panel**). CD4+CD45RO+ memory T cells were sorted into purified populations of hMCAM+ and hMCAM− cells from five individual donors, and stimulated *in vitro* with anti-CD3 and anti-CD28 in the absence of exongenous cytokines (**Figure 1A, right panel**). Although levels of IL17 production varied among donors similar to previous published reports [24], hMCAM+ cells isolated from each of the five donors secreted significantly more IL-17 than hMCAM− cells isolated from the same donor (symbols colored by donor). On average, hMCAM+ cells produced 10-fold more IL17 than hMCAM-negative cells, with hMCAM-negative cells from two of the five donors producing no detectable IL17 secretion. Thus, the overwhelming majority, and in some donors, the totality, of IL-17 production potential among CD4+ T cells was contained within the small population of circulating T cells expressing hMCAM. Nearly 90% of hMCAM expressing T cells freshly isolated from the circulation were CCR6+ and CCR7 negative or low (**Figure 1B**). Further analysis demonstrated that MCAM expression was consistent with that of standard TH17 cell markers: It was limited to CCR6+ cells, expressed on both CD161+ and CD161− cells, and enriching for cells that produce IL17 (**Figure S1**). By these criteria, hMCAM may actually represent a superior marker for circulating TH17 than either CCR6 or CD161, since it defines a much smaller subset of T cells, while identifying the majority of cells capable of making IL17.

To confirm the finding that IL17 was being produced by the hMCAM+ cells from the circulation, we repeated the experiment and looked at intracellular staining for both IL17 and IFNγ after four days of *in vitro* stimulation. While both hMCAM+ and hMCAM− T cells produced comparable amounts of IFNγ, intracellular IL-17 was nearly absent within the hMCAM− population of T cells (**Figure 1C**). It is interesting to note that after 4 days of stimulation about 40% of the hMCAM+ cells lost, or had decreased levels of hMCAM surface expression, while hMCAM− cells remained both hMCAM and IL-17, negative (**Figure 1D**).

### MCAM expressing T cells are expanded by IL1β and produce the majority of both IL-17 and IL-22 under TH17 conditions

**Figure 2:**
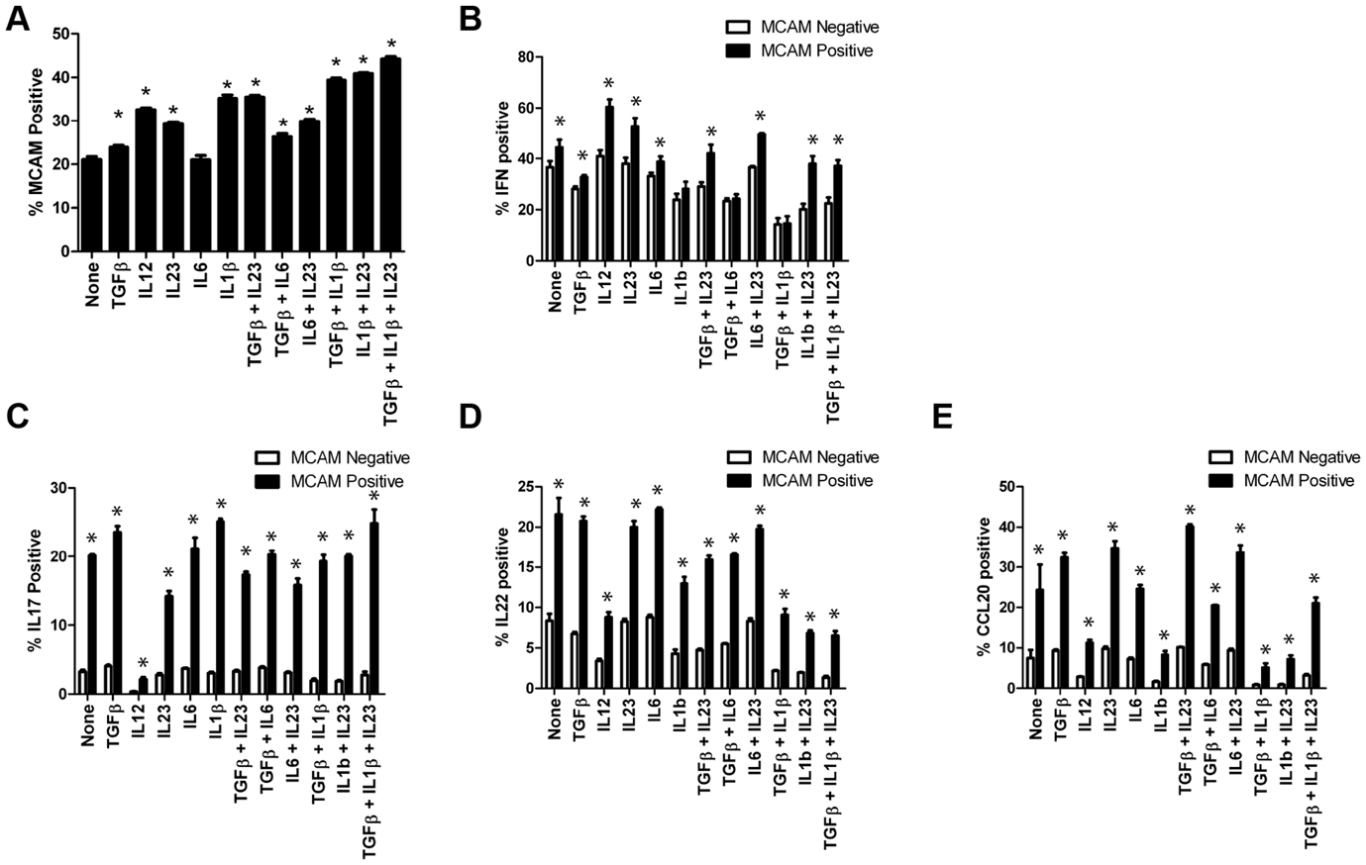
hMCAM+ T cells expand in response to cytokine stimulation, and specifically produce the majority of TH17 cytokines. *(A)* Human CD4+CD45RO+ T cells were stimulated with anti-CD3 and anti-CD28 in the presence of the indicated cytokine(s). Cells were collected after five days, following PMA/Ionomcycin stimulation with golgi inhibition for the final five hours. Cells were stained for surface hMCAM and the percent hMCAM+ is shown (n = 4). Cells, were also stained for intracellular IFNγ (B) IL-17 (C) IL-22 (D) CCL20 (E) following cytokine stimulations and the percent cytokine positive within either the hMCAM− or hMCAM+ cell population is shown (n = 4 for each cytokine analysis). Data is representative of at least five individual donors. * indicates p<0.05.

It has previously been reported that hMCAM positive cells produce IL17 [14], and that there are elevated numbers of hMCAM+ T cells in the circulation of patients with autoimmune disease [25]. To extend these findings to additional TH17 cytokines, and to better understand the regulation of hMCAM expression on the surface of cells, we examined purified human CD4+CD45RO+ T cells stimulated *in vitro*. The cells were exposed to anti-CD3 and anti-CD28 for four days under a number of cytokine conditions, and the percentage of hMCAM expressing cells, as well as cytokine expressing cells was determined by flow cytometry. When introduced as individual cytokines, or in various combinations during stimulation, TGFβ, IL12, IL23 and particularly IL1β all expanded MCAM expression, whereas IL6 had no effect (**Figure 2A**). Both hMCAM+ and hMCAM− cells produced the TH1 cytokine IFNγ(**Figure 2B**), whereas under all stimulatory conditions, hMCAM+ cells produced approximately 5-fold higher levels of IL17 than hMCAM− cells (**Figure 2C**).

The IL-22 receptor is largely expressed on non-immune cells such as epithelial cells and functions in anti-microbial responses as well as tissue remodeling [26], [27]. As with IL-17, IL-22 was expressed preferentially within hMCAM+ cells, though the cytokine mediated regulation of IL-22 differed substantially from that of IL-17 (**Figure 2D**). Differential regulation of IL-22 and IL-17 has been noted previously, [28] and may underscore the myriad functions that hMCAM+ T cells may possess depending upon the cytokine milieu encountered at the site of antigen presentation.

TH17 cells have also been reported to express CCL20, and similar to IL-17 and IL-22, CCL20 expression was more prevalent in hMCAM+ than hMCAM− T cells (**Figure 2E**) [29]. These results suggest a possible positive feedback loop in the migration of CCR6+ TH17 cells, where their release of CCL20 during infiltration likely attracts additional CCR6+ T cells [30]. Thus, hMCAM is not simply a surface marker predicting the capacity of the cell to produce cytokines, but specifically TH17 cytokines.

### Laminin 411, an ECM protein expressed at vascular sites for T cell infiltration, is identified as a major tissue ligand for MCAM

**Figure 3:**
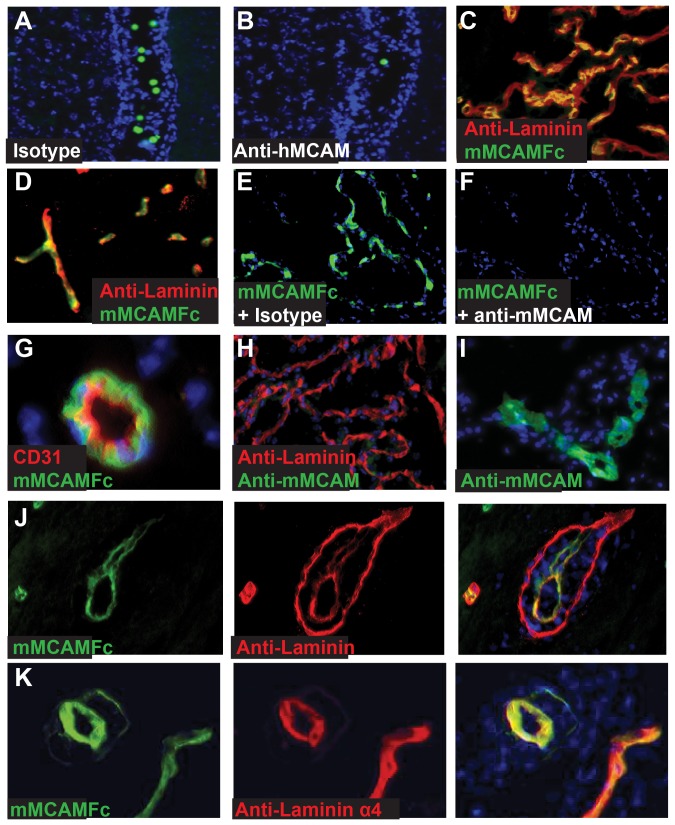
hMCAM binds to a ligand in the ECM with identical staining to laminin α4. Calcein labeled, hMCAM expressing MOLT 4 cells were preincubated with either isotype control (A) or anti-hMCAM (clone 17) (B) followed by incubation on tissue sections from healthy mice. After gentle washing of unbound cells, and mounting with DAPI, bound cells were visualized by fluorescent microscopy. Healthy mouse brain sections containing choroid plexus were stained with fluorescently labeled mMCAM-Fc protein and pan-laminin antibody. Staining of mMCAM-Fc was detected on choroid plexus (C) as well as the vasculature throughout the tissues (D). Fluorescently labeled mMCAM-Fc protein was preincubated with either isotype control (E) or anti-mMCAM (clone 15) (F) before addition to tissue sections of healthy mouse brain. Healthy mouse tissues were stained with fluorescently labeled mMCAM-Fc and CD31 (G) anti-mMCAM and pan laminin (H) or anti-mMCAM alone (I). Tissues from mice with active EAE were stained with fluorescently labeled mMCAM-Fc and pan-laminin (J) or mMCAM-Fc and an antibody specific to the α4 chain of laminin (K).

A function for hMCAM has been elucidated in tumor models, where hMCAM expression confers an adhesive, infiltrative and ultimately metastatic phenotype to tumor cells. hMCAM has been reported to be both a homophilic and a heterophilic adhesion molecule; however, to the best of our knowledge, a homophilic interaction has not been characterized at the biochemical level with purified receptors, and the heterophilic ligand in mammalian cells has not been identified [16], [31]. Therefore, we sought to determine if an MCAM ligand is expressed in the healthy CNS by looking at *ex vivo* cell adhesion with a modified Stamper-Woodruff assay. Initially, we selected the MOLT4 cell line (T cell lymphoma) for these studies, confirming the high level hMCAM expression reported in the literature (FACS detection with anti-MCAM more than 10-fold above isotype control, data not shown). Interestingly, the cells bound selectively to the choroid plexus (**Figure 3A**), an area thought to be important for TH17 cell entry. In looking at a number of anti-human MCAM antibodies, we found this interaction to be inhibited by the antibody clone 17 (**Figure 3B**). These results demonstrated the potential for physiologically-relevant, MCAM−dependent adhesive interactions within the CNS.

To probe the identity of the ligand, we generated an hMCAM-Fc fusion protein to use as a histological reagent on frozen sections of brain and spinal cord. As with the adhesion assay, hMCAM-Fc prominently stained the choroid plexus (**Figure 3C**), and in addition stained blood vessels throughout the CNS (**Figure 3D**). hMCAM-Fc staining of both the vasculature and choroid plexus exhibited a staining pattern similar, but not identical, to that of a pan-laminin antibody. hMCAM-Fc binding to these structures was completely inhibited by the clone 17 antibody against MCAM, confirming the specificity of the interaction (**Figure 3E and 3F**). Vascular staining was localized to the parenchymal side of CD31 expressing endothelial cells within the vasculature, in a manner consistent with that of an extracellular matrix (ECM) protein [32] (**Figure 3G**). Using several different antibodies against mMCAM, we found no apparent staining for mMCAM itself in the choroid plexus (**Figure 3H**), despite occasional staining of vascular endothelial cells in different areas of the same tissues (**Figure 3I**). These results indicate that the recombinant receptor is not interacting in a homophilic fashion with mMCAM in the tissues, and instead suggest an ECM-based ligand for MCAM that is expressed widely in the healthy CNS at potential sites of TH17 cell entry [12], [16]. The results with the recombinant receptor are also consistent with the cell-based observations above, demonstrating the selective adhesion of T cells to the choroid plexus in an MCAM-dependent manner (**Figure 3A**).

In regions of lymphocyte infiltration in EAE, it has been noted that the basement membrane separates into two layers, the endothelial basement membrane and the parenchymal basement membrane, with important distinctions in laminin isoform composition [17]. In sections of EAE brain, hMCAM-Fc stained the endothelial basement membrane, but not the parenchymal basement membrane (**Figure 3J**). This expression pattern has been noted for the 411 isoform of laminin, while both membranes are stained by a pan laminin antibody [18]. Expression of the MCAM ligand colocalized with a laminin 411 (α4 chain) specific antibody (**Figure 3K**) suggesting that laminin 411 might be a specific ligand for MCAM.

**Figure 4:**
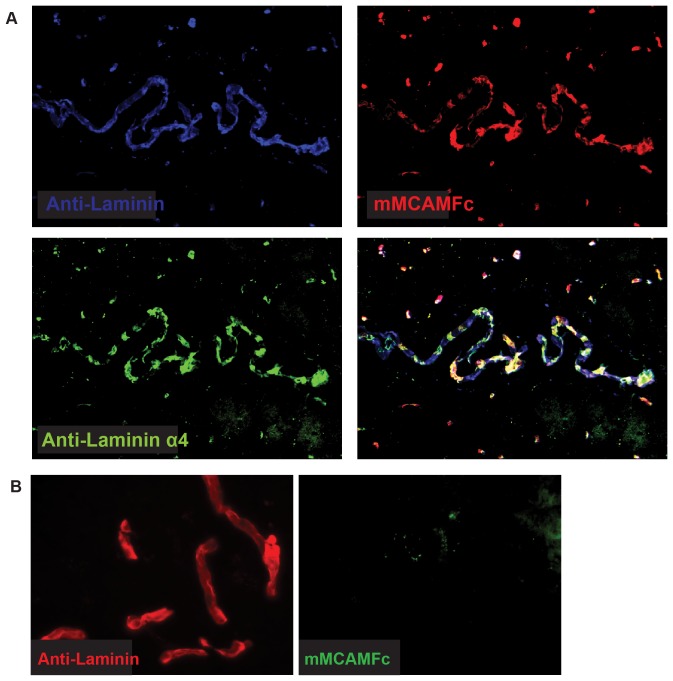
mMCAM colocalizes with laminin 411 on the choroid plexus, and shows no specific binding to tissues from LAMA4−/− mice. *(A)* Staining of healthy mouse choroid plexus with anti-laminin, anti-laminin α4 and mMCAM-Fc (B) Staining of healthy brain tissues from LAMA4−/− mice with anti-laminin and mMCAM-Fc.

These results were also supported by the detailed co-localization of hMCAM-Fc and anti-laminin 411 on the choroid plexus in sections of healthy brain tissue (**Figure 4A**). Finally, the hMCAM-Fc protein did not show specific binding to tissues from laminin α4 deficient mice, indicating that laminin 411 is an important, if not the major tissue ligand for MCAM (**Figure 4B**).

**Figure 5:**
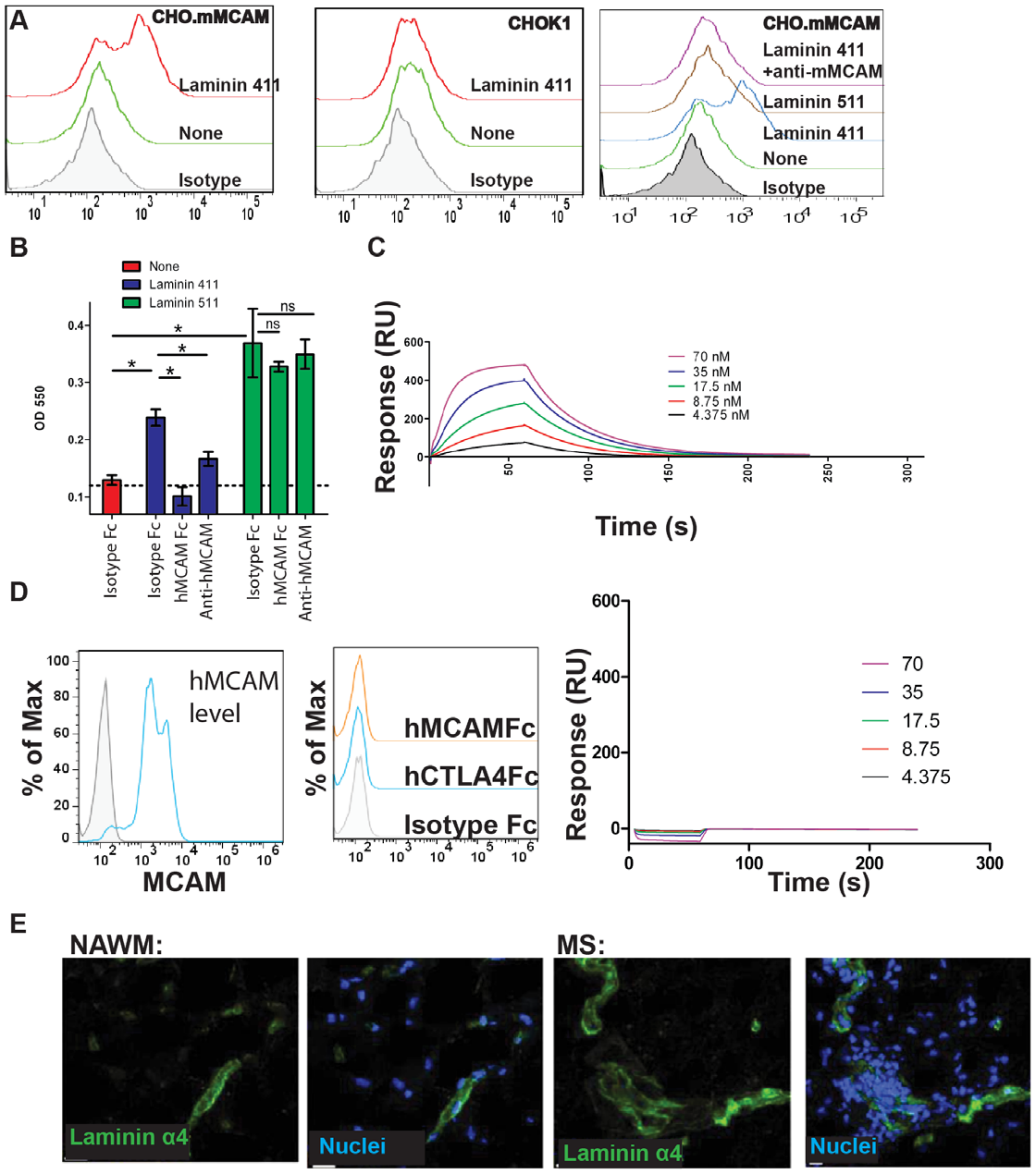
Laminin 411 functions as a ligand for MCAM. (A) CHO cells transfected with hMCAM were incubated with recombinant laminin 411. Laminin binding to the surface of the cells was detected with an anti-laminin antibody (left panel), while no binding was detected to CHO cells lacking MCAM expression (center panel). Recombinant laminin 511 did not bind to MCAM expressing CHO cells, and binding of laminin 411 was specifically inhibited by preincubation of the cells with anti-hMCAM (clone 17, right panel). (B) Human CD4+CD45RO+ memory T cells were purified and incubated with TCR stimulation in the presence of TGFβ and IL1β to induce hMCAM expression (approximately 50% expressed hMCAM, similar to Figure 2A). Cells were incubated in plates coated with either laminin 411 or laminin 511 in the presence of either hMCAM-Fc or anti-hMCAM and specific binding of the cells to the laminin was determined. * indicates p<0.05. (C) Binding of recombinant human laminin 411 to immobilized hMCAM-Fc protein was detected by Biacore with a Kd of approximately 27 nM. Data is representative of three individual experiments. (D) mMCAM is expressed at high levels in nearly all CHO cells transfected with the mMCAM gene, and sorted for high expressors (blue histogram compared to shaded isotype control). However, soluble mMCAM protein does not bind to these cells at levels higher than either human IgG control or irrelevant Fc tagged protein, CTLA-4. Furthermore, no specific binding of mMCAM-His to mMCAM-Fc was detected by Biacore. (E) Laminin α4 was detected in both human MS lesions and normal appearing white matter.

To further explore the functional interaction between MCAM and laminin 411 we examined the ability of mMCAM-expressed cells to capture recombinant laminin 411 from solution. mMCAM-transfected CHO cells were incubated with laminin 411 washed and then exposed to a pan laminin antibody to detect binding of laminin to the surface of the cells. We found that the mMCAM-transfected CHO cells, but not parental CHO cells, captured laminin on their cell surface, and this interaction was inhibited by anti-mMCAM clone 15 (**Figure 5A**). hMCAM-transfected CHO cells showed similar binding to recombinant laminin 411, which was inhibited by anti-hMCAM clone 17 (data not shown). Furthermore, laminin 511 which shares the same β and γ chain with laminin 411, differing only in the α5 vs. α4 chain subunits did not bind to MCAM-transfected CHO cells. These experiments demonstrate that MCAM specifically interacts with laminin 411 since the cell surface interaction is dependent upon MCAM expression, is inhibited by an MCAM antibody, and does not occur with laminin 511.

To expand upon the potential cell adhesive function of this interaction we examined CD4+CD45RO+ T cells. Human CD4+CD45RO+ T cells were isolated from the circulation and stimulated with IL-1β to expand MCAM+ cells (approximately 50% of the cells were hMCAM+). When exposed to plated substrate, the primary T cells (**Figure 5B**) bound to both laminin 411 and laminin 511. Adhesion to laminin 411 was fully disrupted in the presence of hMCAM-Fc and also to a large extent by the anti-hMCAM antibody (clone 17 anti-MCAM). In contrast, adhesion to laminin 511 was not affected by either reagent. These results demonstrate that MCAM supports cell adhesion to laminin 411, and that adhesion to laminin 511 is distinct. The superior blockade of adhesion to laminin 411 by the hMCAM-Fc protein may represent the ability of the soluble hMCAM construct to bind laminin 411 and neutralize binding sites for hMCAM as well as for other laminin-binding adhesion molecules expressed by the cells, including integrins α6β1 or α3β1 [33], although this idea needs to be addressed with well defined integrin-dependent adhesion assays.

To further characterize the novel interaction between MCAM and laminin 411, we examined the interaction by surface plasmon resonance (SPR). Soluble laminin 411 exhibited a direct interaction with immobilized hMCAM, with an apparent KD of 27 nM, (**Figure 5C**). We were unable to detect an interaction between laminin 511 and hMCAM using the same approach, further confirming that the interaction was specific to the laminin 411 isoform (**Figure S2A**). Furthermore, interaction between laminin 411 and hMCAM was entirely blocked in the presence of anti-hMCAM antibody clone 17 (**Figure S2B**), indicating the specificity of the interaction. Thus, MCAM is a bona fide binding partner for laminin 411. With this very sensitive system of SPR, we were unable to detect an interaction of MCAM with itself (human or mouse, data not shown), nor could we detect interaction between hMCAM-Fc and hMCAM transfected CHO cells (**Figure 5D**). While it remains possible that cell surface MCAM can adopt a conformation permissive for homotypic adhesion in some systems, it is clear that MCAM-Fc does not support homophilic interactions with itself or with cell surface MCAM. These data, however, strongly support a heterophilic interaction between MCAM with Laminin 411, and along with the knock out data above, indicate that laminin 411 is a major tissue ligand for MCAM. To confirm that laminin 411 is expressed in human brain vessels, brain tissue from both control and MS patients was exposed to an antibody against laminin 411. Similar to the mouse, laminin α4 was widely expressed in the normal appearing white matter in both MS patients and healthy donors (**Figure 5E**).

### MCAM is induced on mouse T cells with TH17 polarization, and MCAM blockade inhibits EAE disease progression

We generated antibodies against murine MCAM and confirmed their MCAM specific binding with cell transfection experiments and FACS analysis, and then screened for those that could block the ability of murine MCAM to bind laminin 411. The best murine-specific, laminin-inhibitory antibody was clone 15. While the anti-mouse MCAM antibodies identified a population of NK cells in the circulation of mice, there was no staining of circulating mouse T cells (data not shown). These results are consistent with a previous report using an independent anti-mouse MCAM antibody [34].

In humans, since MCAM is expressed primarily on memory T cells in the circulation, we considered the possibility that laboratory mice, living in a clean environment with limited previous T cell activation, may lack such an antigen-experienced population of T cells. Therefore, we stimulated mouse T cells in cell culture under TH17 polarizing conditions to determine if MCAM could be induced upon polarization, as seen with human cells above.

**Figure 6:**
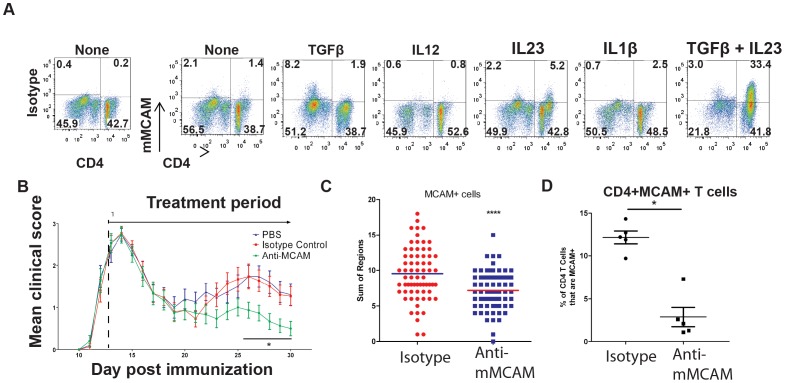
Mouse T cells express MCAM following TH17 polarization, and blockade of MCAM influences disease progression in EAE. *(A)* A population of PLP specific T cells was generated *in vivo* by immunization of SJL mice with PLP in CFA. Splenocytes from immunized mice were restimulated *in vitro* with PLP in the presence of the indicated cytokine(s), and mMCAM expression on CD4+ T cells was determined. (B) SJL mice were immunized with PLP/CFA as described. Two days after onset of disease, (between days 12 and 14 post immunization), and daily thereafter, the animals received either anti-mMCAM (clone 15) neutralizing antibody or isotype control antibody. Disease progression was scored, and body weights were monitored. * indicates p<0.05 by Wilcoxon's non-parametric test. Data represents the mean of 15 mice ± sem. Quantification of infiltrating mMCAM+ cells (C) from EAE induced mice treated with either isotype control or anti-mMCAM. **** indicates P<0.0001. Sections were scored as described in Methods S1. (D) T cells were isolated from the CNS of mice treated with either isotype control or anti-mMCAM and analyzed by flow cytometry for expression of CD4 and mMCAM. Graph indicates the percentage of CD4+ cells that are mMCAM+ in either treatment group. Each dot represents the percentage of mMCAM+ cells within the CD4+ T cell population in a single mouse. * indicates p<0.05.

Splenocytes from PLP immunized mice were restimulated with PLP *in vitro* under various cytokine conditions. After five days in culture, the cells were analyzed by FACS for mMCAM expression (**Figure 6A**). In the absence of exogenous cytokines, restimulation induced mMCAM expression on a small population of CD4+ cells (as well as CD4− cells) compared to isotype control antibody. This population was enhanced in the presence of IL-23, and although TGFβ had only a small impact on its own, synergy between TGFβ and IL-23 generated mMCAM expression among nearly half of CD4+ T cells. Both of these cytokines have a critical role in the polarization and effector function of mouse TH17 cells [35], [36]. Notably, mMCAM was expressed solely on a population of CD4 high T cells which have been described to contain the pathogenic T cells in EAE [37].

Having confirmed the potential for mouse T cells to express MCAM under TH17 polarizing conditions, we examined the effects of mMCAM blockade on progression of disease in a therapeutic model of EAE, where such polarization could occur *in vivo*. Disease progression was monitored following PLP immunization, and two days after the onset of clinical symptoms (typically day 12 or 13 post immunization) animals received daily treatment with a mouse mMCAM specific antibody, clone 15 or isotype control antibody. Treatment began near the peak of the acute phase of disease, which resolved along a normal course in both control and anti-MCAM treated animals. However relapse was delayed and significantly less severe in mice treated with anti-mMCAM (**Figure 6B**). Likewise, animals treated with anti-mMCAM gained more body weight than control-treated EAE animals (**Figure S3A**, n = 15/group, blinded observer). Histological analysis of tissues taken from all animals at the end of the study (day 30) showed that, compared to isotype control, anti-mMCAM treatment resulted in significant decreases in the number of infiltrating mMCAM+ T cells (**Figure 6C**) and CD4+ T cells (**Figure S3B**). It also decreased inflammatory demyelination (**Figure S3C**). The EAE experiment was repeated (with similar efficacy by anti-MCAM vs. isotype antibody), and infiltrating cells were isolated from the brains and spinal cords of five animals from each group at the peak of relapse (day 25 post immunization) for FACS analysis. This analysis confirmed that the mice generated CD4+MCAM+ T cells *in vivo*, representing approximately 12% of the infiltrating CD4+T cells in control-treated EAE animals, and showed that treatment with anti-mMCAM reduced this percentage by more than 80% (**Figure 6D**; as measured by labeling with an anti-mMCAM antibody that is not competed by the therapeutic, mouse MCAM specific, clone 15 anti-MCAM antibody). Furthermore, to confirm expression of IL17 within murine MCAM expressing T cells, splenocytes from PLP immunized mice were restimulated in vitro in the presence of TGFβ and IL23, and stained the cells for surface expression of MCAM using anti-mMCAM antibody, clone 15 (versus an isotype control for determining gates), and intracellular IL-17. Greater than 70% of the IL-17 producing CD4+ T cells expressed MCAM (**Figure S3D**), confirming that a similar linkage between MCAM and IL-17 exists in mice as we describe for human T cells. These results with an inhibitory antibody against mMCAM in mouse EAE are consistent with the idea that MCAM+ CD4 T cells are involved in promoting the inflammatory disease process.

Identification of MCAM on the surface of TH17 cells, and the discovery of laminin 411 as a receptor for MCAM, define a molecular interaction that could contribute to the unique ability of TH17 cells to infiltrate the CNS, as well as other tissues.

## Discussion

During multiple sclerosis, lymphocytes gain entry to the CNS and mount a pathogenic autoimmune response against myelin antigens [38]. The role of T cells, and specifically the involvement of TH17 cells, has been well characterized, though the precise molecular determinants are unclear [39]. A suggestion arising from mouse models, is that a distinct subpopulation of CCR6+ TH17 cells infiltrate the CNS at several possible anatomical locations, including the choroid plexus, and upon recognition of cognate antigen, facilitate a more promiscuous T cell response, including non-TH17 cells.

Models postulating that TH17 cells have a prominent role in promoting inflammation presuppose that such T cells possess unique migratory properties for tissue entry. While multiple adhesive and chemokine pathways have been identified that contribute to this process, none are unique to TH17 cells [40], [41].

Reboldi *et al* reported that CCR6 expression is required for the initial wave of T cells to migrate to the choroid plexus, where CCL20 is expressed [42]. In the absence of a CCR6 expressing T cell population, disease progression in EAE is significantly ameliorated. We discovered that there is a subpopulation of CCR6 expressing T cells in the circulation that co-express MCAM, and account for the majority of IL-17 production in ex vivo analysis. Importantly, following ex vivo activation, nearly all IL-17 is secreted from CD4+CD45RO+MCAM+ T cells.

MCAM expression has been well studied in melanoma, and MCAM expression on tumor cells appears to confer metastatic potential onto non-metastatic cells [13]. When one considers the process of metastasis, and its parallels to the vascular dissemination of T cells into tissues, it is reasonable to consider that MCAM expression could contribute to the unique infiltrative capacity of TH17 cells.

We were surprised to find extensive but specific binding of MCAM-Fc to the vasculature ECM in all organs analyzed, and were able to identify the specific binding partner for MCAM as laminin 411, an isoform of laminin expressed by vascular endothelial cells. The distribution of laminin 411 as an MCAM ligand suggested that it could confer wide access to the small subset of MCAM+ T cells.

In that regard, it is interesting that the circulating MCAM+ population exists nearly exclusively within the CD45RO+ memory T cells under homeostatic conditions. Memory T cells have the unique ability to migrate directly into tissues and be reactivated by non-professional antigen presenting cells due to their lower threshold for T cell stimulation [43], [44]. This reactivation step is likely critical to ongoing pathogenesis in EAE, and since it occurs within the CNS, rather than an organized lymphoid tissue, it seems reasonable that a population of antigen-specific memory T cells would be involved.

While EAE has been extensively used as a model for MS, discrepancies with the human disease are commonly discussed in the literature [45]. Notable differences between mouse and human TH17 cells have vexed the field for some time, particularly in regard to the cytokines required for polarization of IL-17 producing CD4+ T cells [46]. With T cells isolated from the circulation of humans, nearly all IL17 production that occurs with *in vitro* stimulation in the absence of polarizing cytokines emanates from CD45RO+ memory T cells that also express MCAM. In contrast, such a memory population of T cells may not exist in young mice raised in clean environments, which are almost invariably the mice used in experiments. These differences might explain our data indicating that while TGFβ is required for MCAM expression on mouse T cells, it is not required to generate or expand MCAM expressing T cells in humans, where there is a pre-formed population of MCAM+ memory TH17 cells. These differences may also explain why inhibition of IL-23, a cytokine required in the early stages of TH17 cell differentiation, is more effective in mouse models of EAE than in human studies of MS [47].

The identification of laminin 411 as a ligand for MCAM provides the necessary binding partner to support the hypothesis that MCAM could contribute to the infiltrative capacity of TH17 cells into the CNS. The fact that MCAM, a five Ig domain protein, binds to laminin is consistent with previous data showing that an avian homolog of MCAM, gicerin, binds to neurite outgrowth factor, a homolog of laminin [48]. Furthermore, B-CAM, another 5 Ig domain protein with some homology to MCAM functions as a receptor for laminin 511 [49]. Although a report suggests that MCAM expression on tumor cells decreases the adhesion of the cells to laminin, the composition of the laminin in that study is unclear, and may have lacked the laminin 411 isoform [50], [51]. Other studies have linked expression of MCAM on tumor cells to their metastatic potential [52].

It has been previously reported that laminin 411 is important for T cell infiltration of the CNS during EAE. Laminin 411 is expressed at sites of T cell entry in the CNS, including the choroid plexus and postcapillary venules, and mice deficient in laminin 411 display diminished disease in EAE [17], [18], [53]. T cell transmigration into the CNS occurs in regions where laminin 411 is present, but laminin 511 is absent. It was proposed that α6β1 integrin functions as the receptor for laminin 411. However, as noted by the authors of the study, α6β1 integrin is expressed on a large population of T cells and, combined with the ubiquity of laminin 411 expression, makes it difficult to reconcile how a6β1 alone could confer specific migratory capacity. While part of this selectivity for laminin 411 may be explained by the apparent inhibitory effect of laminin 511 on integrin α6β1 mediated binding *in vitro*, an alternate explanation is that MCAM adhesion to the laminin 411 isoform contributes to this process, working either alone or in conjunction with α6β1 integrin. The limited expression of MCAM within a small population of highly pathogenic memory T cells could lend a greater degree of selectivity to the system.

The findings presented in this study provide compelling evidence that MCAM functions as an adhesion molecule on circulating TH17 cells to facilitate their entry into the CNS (and likely other tissues, including skin [14]). These finding include: 1) selective expression of MCAM on 3–5% of circulating CD4+ T cells, all within in the memory population; 2) MCAM+ T cells isolated from the circulation account for nearly all of the IL17 production when stimulated *in vitro* without specific TH17 polarization; 3). The major tissue ligand for MCAM is laminin 411, which is selectively expressed within the tissue vasculature; 4). Inhibition of MCAM with a specific antibody, or of laminin 411 with MCAM-Fc, blocks T cell adhesion to laminin 411 in vitro. 5) Blockade of the laminin binding activity of MCAM in vivo reduces MCAM+ T cell infiltrates in the brain and reduces disease progression in EAE. Furthermore, these results are consistent with genetic data by Wu, et al., showing that knock out of laminin 411 inhibits CD4+ T cell infiltration of the CNS and reduces disease in EAE [18], and supports previous literature implicating MCAM in cell migration within the vasculature.

The specific location of laminin 411 in the endothelial basement membrane may either function to augment adhesion of cells attempting CNS endothelial penetration, or serve as an adhesion-based gating system to signal appropriate entry mechanisms, adding another element of regulation and selectivity to other adhesion and signaling molecules within the vascular adhesion and migration cascade. As such, modulation of the interaction between MCAM and laminin 411 represents a novel and selective approach that may help to maintain or restore homeostasis to inflamed tissues in autoimmune diseases.

## Supporting Information

Figure S1 **Expression of hMCAM and IL17 relative to known TH17 markers, CCR6 and CD161.** Human CD4+ T cells were isolated and stimulated for five hours with PMA/Ionomycin and golgi inhibition before staining for CCR6, CD161, hMCAM and intracellular IL17. Top row shows the percentage of hMCAM+ cells within each of the four permutations of CCR6 and CD161. Middle row shows the percentage of IL17 positive cells within the four permutations of CCR6 and CD161. Bottom row shows 3–4 fold enrichment of IL17 within the hMCAM+ population of either CCR6+CD161− or CCR6+CD161+ cells. Consistent with Figure 2B, hMCAM was nearly absent within CCR6− cells, (there was a small presence within the CCR6−CD161+ cell population). Likewise, IL17 expression was limited to CCR6+ cells, with some further enrichment based on CD161 expression. Gating on these populations separately, it is clear that hMCAM was enriched 3–4 fold in either the CCR6+CD161− or the CCR6+CD161+ populations.
(TIF)

Figure S2 **hMCAM does not bind to laminin 511, and binding of hMCAM to laminin 411 specificity is confirmed in the presence of neutralizing anti-hMCAM antibody.** *(A) h*MCAM-Fc was immobilized, and binding of laminin 511 by itself (B) or laminin 411 in the presence of anti-hMCAM neutralizing antibody (C) was measured. Data is representative of three individual experiments.
(TIF)

Figure S3 **Additional data on antibody efficacy in EAE.** (A) Body weights * indicates p<0.05 by Wilcoxon's non-parametric test. (B) Quantification of CD4+ T cells and (C) demyelination scores from the IHC of mice in EAE study as described in Figure 6. ** indicates p<0.01. Sections were scored as described. (D) Mice were immunized with PLP as described in Materials and Methods. After 11 days, spleens were removed, and RBC depleted splenocytes were re-stimulated in vitro with PLP (5 µg/ml), TGFβ (5 ng/ml) and IL-23 (20 ng/ml). After six days incubation, cells were collected and cell surface was stained with CD4 and either anti-mMCAM (clone 15) or an appropriate isotype control, followed by staining for intracellular IL-17.
(TIF)

Methods S1 Supplementary methods.
(DOCX)
