# Supplementary figures and images for: Laminin-411 Is a Vascular Ligand for MCAM and Facilitates TH17 Cell Entry into the CNS
Source: PLoS One. 2012 Jul 6;7(7):e40443. doi: 10.1371/journal.pone.0040443 (PMC3391262; doi:10.1371/journal.pone.0040443)

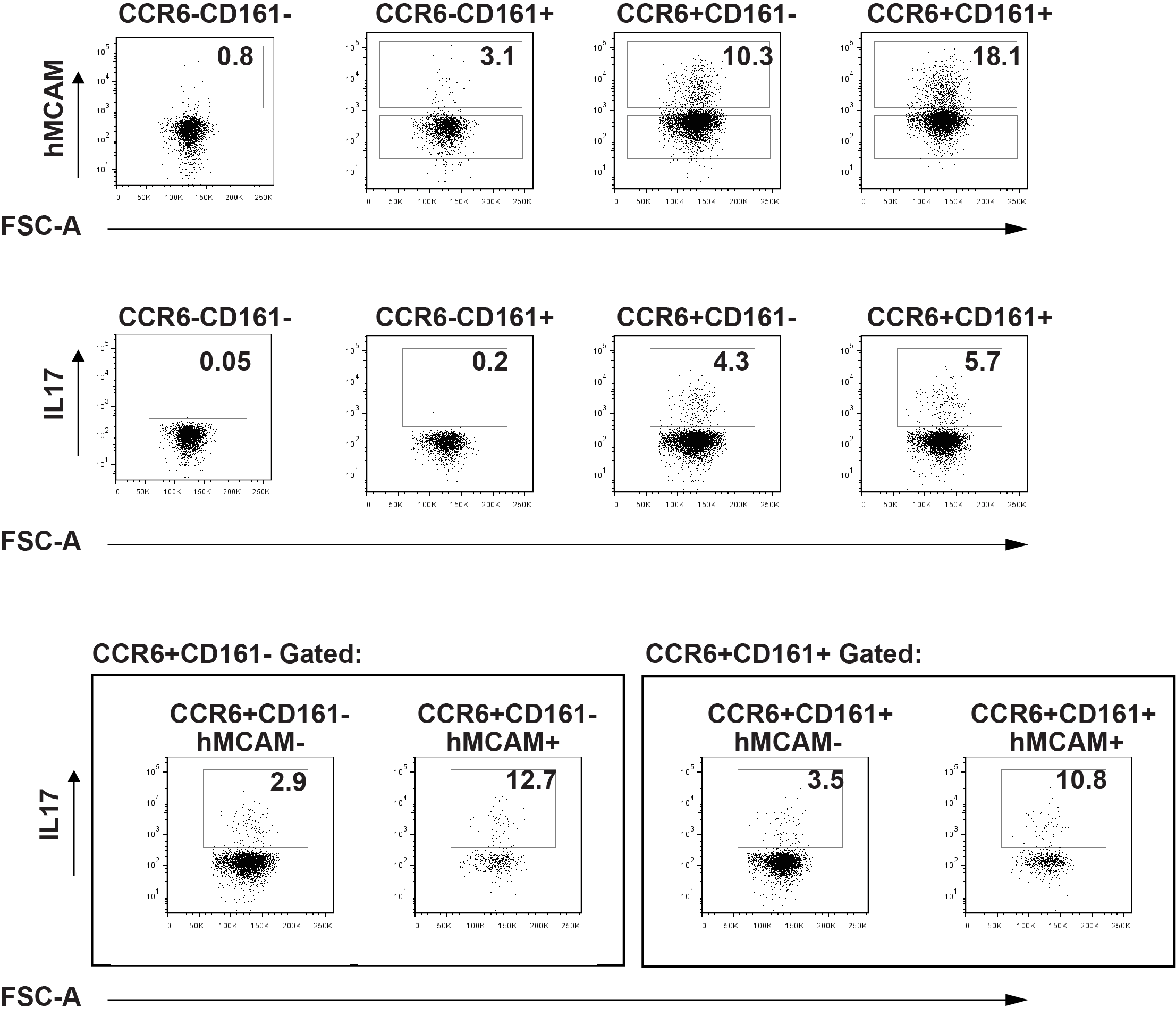

Supplement: Figure S1 — Expression of hMCAM and IL17 relative to known TH17 markers, CCR6 and CD161. Human CD4+ T cells were isolated and stimulated for five hours with PMA/Ionomycin and golgi inhibition before staining for CCR6, CD161, hMCAM and intracellular IL17. Top row shows the percentage of hMCAM+ cells within each of the four permutations of CCR6 and CD161. Middle row shows the percentage of IL17 positive cells within the four permutations of CCR6 and CD161. Bottom row shows 3–4 fold enrichment of IL17 within the hMCAM+ population of either CCR6+CD161− or CCR6+CD161+ cells. Consistent with Figure 2B, hMCAM was nearly absent within CCR6− cells, (there was a small presence within the CCR6−CD161+ cell population). Likewise, IL17 expression was limited to CCR6+ cells, with some further enrichment based on CD161 expression. Gating on these populations separately, it is clear that hMCAM was enriched 3–4 fold in either the CCR6+CD161− or the CCR6+CD161+ populations. (TIF) [file pone.0040443.s001.tif]

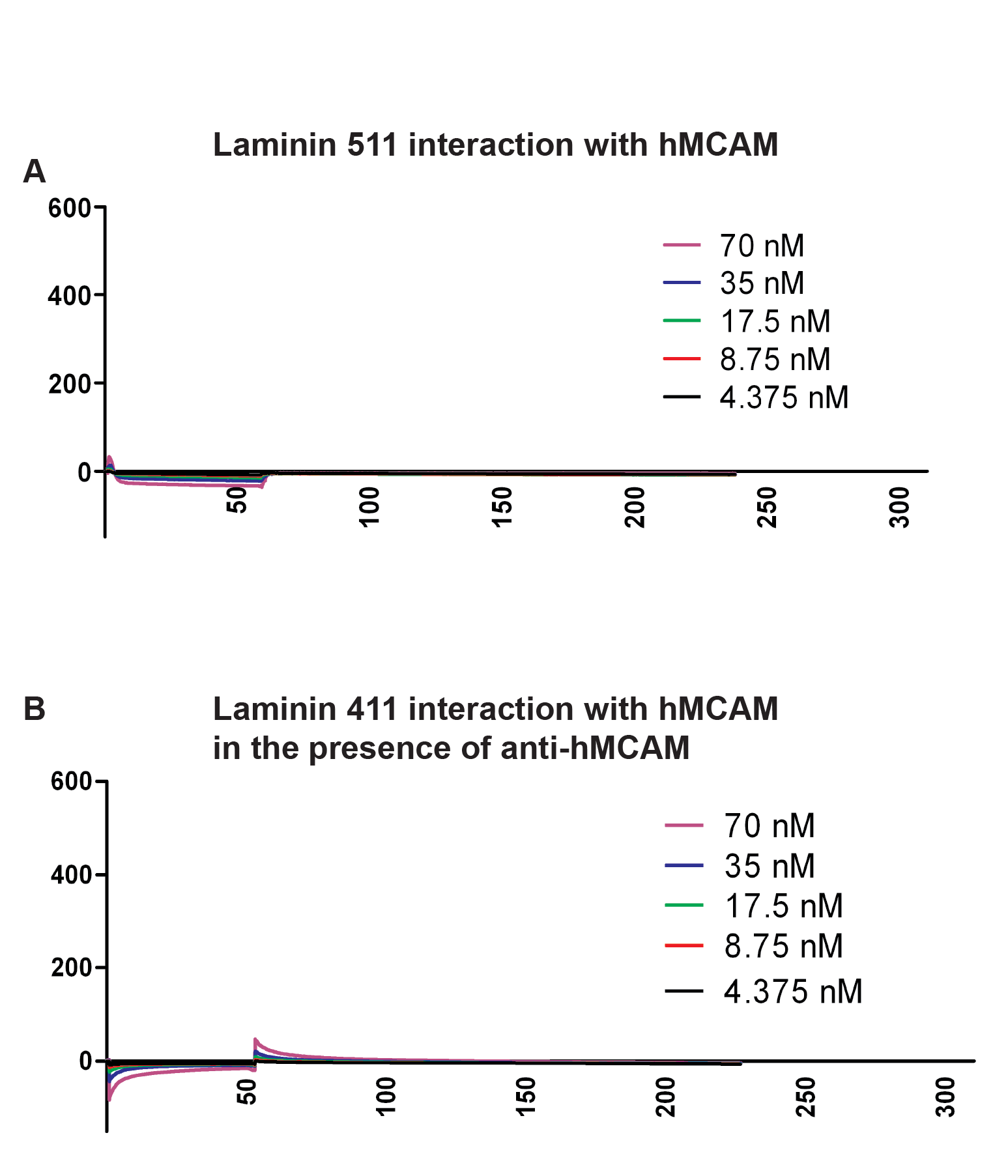

Supplement: Figure S2 — hMCAM does not bind to laminin 511, and binding of hMCAM to laminin 411 specificity is confirmed in the presence of neutralizing anti-hMCAM antibody. (A) hMCAM-Fc was immobilized, and binding of laminin 511 by itself (B) or laminin 411 in the presence of anti-hMCAM neutralizing antibody (C) was measured. Data is representative of three individual experiments. (TIF) [file pone.0040443.s002.tif]

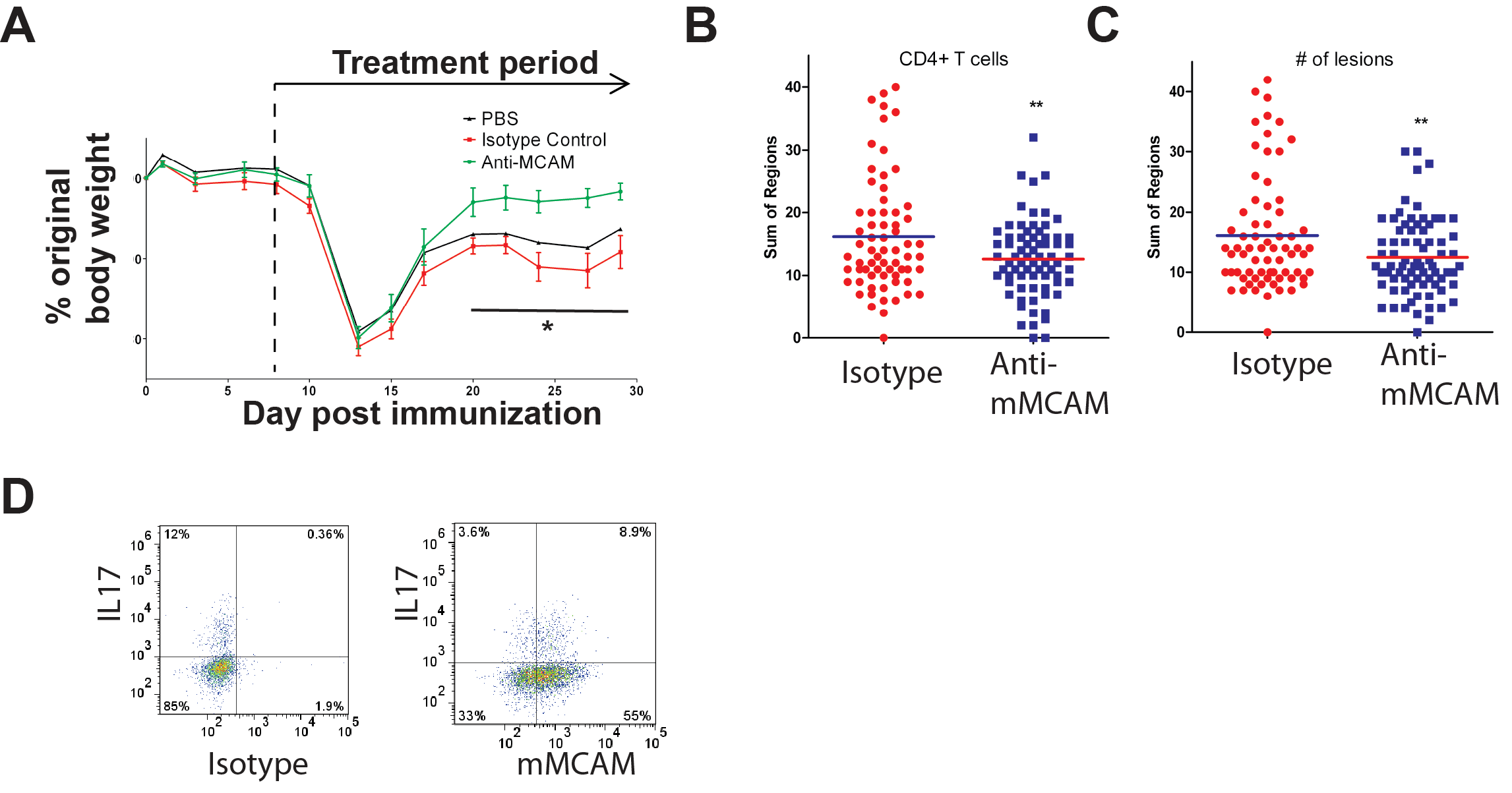

Supplement: Figure S3 — Additional data on antibody efficacy in EAE. (A) Body weights * indicates p<0.05 by Wilcoxon's non-parametric test. (B) Quantification of CD4+ T cells and (C) demyelination scores from the IHC of mice in EAE study as described in Figure 6. ** indicates p<0.01. Sections were scored as described. (D) Mice were immunized with PLP as described in Materials and Methods. After 11 days, spleens were removed, and RBC depleted splenocytes were re-stimulated in vitro with PLP (5 µg/ml), TGFβ (5 ng/ml) and IL-23 (20 ng/ml). After six days incubation, cells were collected and cell surface was stained with CD4 and either anti-mMCAM (clone 15) or an appropriate isotype control, followed by staining for intracellular IL-17. (TIF) [file pone.0040443.s003.tif]
