# Supplementary material for: Laminin-411 Is a Vascular Ligand for MCAM and Facilitates TH17 Cell Entry into the CNS
Source: PLoS One. 2012 Jul 6;7(7):e40443. doi: 10.1371/journal.pone.0040443 (PMC3391262; doi:10.1371/journal.pone.0040443)
Supplement: Methods S1 — Supplementary methods. (DOCX) [file pone.0040443.s004.docx]

**Supplementary Materials and Methods**

*Cells*

CHO cells (ATCC) were cultured in DMEM containing 10% heat-inactivated FCS (HyClone Laboratories), penicillin, streptomycin, L-glutamine. Cells were transfected with full length MCAM and selected for stable expression by neomycin resistance using lipofectamine and standard protocols. Human T lymphoblast cell line, MOLT4, were obtained from ATCC and cultured in RPMI containing 10% FCS. CHO-K1 cells were transfected with the full length murine MCAM gene using lipofectamine, and manufacturer’s protocol. Upon transfection, the cells were selected and maintained in 10 µg/mL puromycin. Flow cytometric analysis confirmed surface murine MCAM.

*Antibodies*

Staining of human cells was performed using the following antibodies: MCAM (P1H12, BD Pharmingen), CD4 (RPA-T4, BD Pharmingen), CD45RO (UCHL1, BD Pharmingen), CD161 (DX12, BD Pharmingen), IL17 (EBIO64DEC17, Ebioscience), IL22 (22URT1, Ebioscience), CCL20 (67310, R&D Systems). Staining of mouse cells was performed using the following antibodies: Isotype control (43414, R&D Systems), CD4 (RM4-5, BD Pharmingen), MCAM (Either clone 15 or 17 as indicated, generated in house), IL-17 (clone TC11-18H10; BD Pharmingen).

*Recombinant protein generation*

MCAMFc protein was generated by fusion of the extracellular domain of the murine MCAM gene to human IgG1 and expression in CHO cells. MCAMFc protein was purified from the supernatant of transfected cells using Mabselect columns (GE Healthcare).

*Antibody generation*

Five-week- old female Lou/M rats were hyperimmunized with 100ug of mouse MCAM.hFc  fusion protein via intraperitoneal pathway with Freund’s complete or incomplete adjuvant.  After three injections, mouse MCAM specific serum titer was  tested by FACS using CHO.mMCAM transfectants.  Three days after the final boost with immunogen via tail vein without adjuvant, splenocytes were isolated from the highest titer rat for fusion with YB2/0 myeloma cells to generate hybridomas using standard polyethylene glycol protocol.  Initial fusion screen was based on binding to native mouse MCAM expressed on CHO cells, counter screening by lack of binding to parental CHOK1 cells, followed by functional screening based on the ability to block specific mMCAM-Fc binding to tissues. Two clones (Clone 15 and Clone 17) emerged from the screen displaying specificity for binding to mMCAM (based on binding to mMCAM transfected CHO cells) as well as inhibition of mMCAM mediated adhesion. The two clones were screened for binding to human MCAM expressing CHO cells, and clone 17 displayed crossreactivity for human MCAM (based on binding to hMCAM transfected CHO cells), while clone 15 was specific for mouse MCAM (based on lack of binding to hMCAM transfected CHO cells). Immunization and euthanasia of rats were performed under ELAN IACUC protocol in keeping with the policies of the N.I.H. Guide for the Care and Use of Laboratory Animals (revised 1985) and OPRR guidelines (revised Sept. 1986). Pain and distress was limited to routine injections, and euthanasia occurred by CO_2_ Narcosis followed by bilateral thoracotomy.

*Flow cytometric analysis*

For flow cytometric analysis, cells were surface stained using the indicated antibody and standard protocols. Where required, cells being stained for intracellular cytokines were stimulated for five hours with PMA (50 ng/ml) and Ionomycin (500 ng/ml) in the presence of Golgi-stop (BD Pharmingen, 1:1500 final). Cells were first surface stained, then fixed and permeabilized using fixation and permeabilization buffer, and manufacturer’s protocols (Ebioscience), followed by intracellular staining with the indicated antibody before analysis by flow cytometry (LSRII, BD Pharmingen).

*Analysis of human tissues by immunofluoruescent staining*

Human brain samples were obtained post-mortem after (ante-mortem) informed written consent from MS patients in accordance with institutional guidelines (CHUM research ethic committee approval number SL05.022 and 023 and BH07.001).
and normal brain tissue was obtained after informed written consent from non-epileptic surgical human CNS material, in accordance with institutional guidelines (CHUM research ethic committee approval number HD04.046).

For human tissues, post-mortem frozen sections (*n* = 4) from brains of 2 RRMS patients and 2 healthy controls (normal brain tissue from non-epileptic surgical human CNS material) were studied as previously described[^1^](#_ENREF_4). Briefly, active MS lesions were identified by LFB and haematoxilin and eosin (H&E) staining and defined as areas of demyelination associated with intense perivascular immune cell infiltration. For immunohistofluorescence, sections were fixed in acetone for 10 minutes and then put in ethanol for 5 minutes, hydrated in PBS and blocked with serum 10% (from the same species that the fluorochrome conjugated antibodies were derived) for 30 minutes at room temperature. Sections were incubated with primary antibodies, washed extensively followed by incubation with appropriate fluorescently conjugated secondary antibodies when necessary. Sections were mounted using gelvatol containing Topro-3 (Invitrogen, 1:300) when required. We used the following primary antibodies: mouse anti-human laminin alpha 4 (monoclonal, 3H2 Santa Cruz Biotechnology).

*Analysis of CNS infiltrating T cells*

Brain and spinal cord from each mouse was pooled, and mechanically disrupted (GentleMACS, Miltenyi Biotec). Cells were passed through a 70 µM cell strainer, washed twice and resuspended in 70% percoll. Cells were overlaid with 37% percoll and spun at 500 g for 20 minutes (brake off). Interphase containing mononuclear cells was collected and washed prior to staining with anti-CD4 and anti-MCAM (clone 17) and analysis by flow cytometry.

*Quantification of clinical scores, and inflammation in EAE samples*

Mice were scored in a blinded fashion as follows:

0 No obvious changes in motor functions of the mouse in comparison to non-immunized mice.

1 Limp tail.

2 Limp tail and weakness of hind legs

3 Limp tail and complete paralysis of hind legs

4 Limp tail, complete hind leg and partial front leg paralysis.

5 Complete hind and complete front leg paralysis, no movement around the cage.

Mouse spinal cords were collected immediately post animal termination by carbon dioxide narcosis. Briefly, the vertibral column was exposed at the cervical and lumbar levels and hydraulically expulsed from the spinal canal. Collected spinal cords were frozen flat prior to embedding in a suitable cryosectioning medium. Spinal cords were segmented into 4-6 anatomical regions and all 5 sections from each animal were embedded adjacent in a rostral to caudal sequence. Coronal spinal cord sections were generated at thickness of 100 um and dry mounted to Superfrost Plus slides. Slides were stored at -20C prior to use.

Stained slides were evaluated according to the criteria outlined below. Briefly, 10 sections per anatomical level (4-6) were individually assigned a score for a total of 40-40 sections evaluated per animal. Scores were summed by anatomical level and plotted by dosing group. Statistical significance was determined by 2-tailed unpaired Students T-Test using a 95% confidence interval.

**MCAM staining (Clone 17)**

0 negative for staining

1 positive for staining (1-9 extravascular cells)

2 More than 10+ extravascular cells

**CD4 staining (R&D Systems)**

0 presence of no more than 4 dispersed cells

1 presence of 4+ dispersed cells or one small cluster

2 1-2 vessels involved with clustering and presence of dispersed cells

3 3+ vessels involved with clustering and presence of dispersed cells or one confluent area

4 2+ confluent areas

**Demyelination (Luxol Fast Blue + H&E)**

1. No demyelination
2. 1-3 small foci of demyelination
3. 4-6 small foci or one small confluent area of demyelination
4. 6+ small foci or one large confluent area of demyelination
5. Several large confluent areas of demyelination
6. >50% of parenchyma is demyelinated

**Statistics**

All analyses were performed by Student’s T test unless otherwise indicated.
